# Is fidelity to a complex behaviour change intervention associated with patient outcomes? Exploring the relationship between dietitian adherence and competence and the nutritional status of intervention patients in a successful stepped-wedge randomised clinical trial of eating as treatment (EAT)

**DOI:** 10.1186/s13012-021-01118-y

**Published:** 2021-04-26

**Authors:** Alison K. Beck, Amanda L. Baker, Gregory Carter, Laura Robinson, Kristen McCarter, Christopher Wratten, Judith Bauer, Luke Wolfenden, Benjamin Britton

**Affiliations:** 1 School of Medicine and Public Health, The University of Newcastle, Callaghan, NSW 2308 Australia; 2 Centre for Brain and Mental Health Research, University of Newcastle, Callaghan, NSW 2308 Australia; 3 School of Psychology, Faculty of Social Sciences, University of Wollongong, Wollongong, NSW 2522 Australia; 4 Department of Radiation Oncology, Calvary Mater Newcastle Hospital, Waratah, NSW 2298 Australia; 5 School of Human Movement and Nutrition Sciences, The University of Queensland, St Lucia, QLD 4072 Australia; 6 Hunter New England Health, New Lambton, 2305 Australia

**Keywords:** Fidelity, Behavioural medicine, Translational research, Implementation science, Motivational interviewing, Behaviour change counselling

## Abstract

**Background:**

Behavioural medicine is characterised by findings for the effectiveness and efficacy of complex behaviour change interventions. Comparatively, scant attention has been paid to key intervention components or mechanisms of action. Evaluating relationships between process variables (fidelity) and intervention effects is central to addressing this imbalance. Accordingly, in the current study, we sought to explore the magnitude and direction of effect between fidelity predictors (dietitian adherence and competence) and intervention effects (patient nutritional status) during the intervention phase of a real-world, stepped-wedge evaluation of ‘EAT: Eating As Treatment’.

**Methods:**

The EAT clinical trial was conducted within five major Australian hospitals located in Queensland, Western Australia, Victoria and South Australia between 2013 and 2016. EAT is a dietitian-delivered health behaviour change intervention designed to reduce malnutrition in head and neck cancer (HNC) patients undergoing radiotherapy. Dietitian adherence and competence ratings were derived from a 20% random sample of audio-recorded dietetic consultations (*n*=194) conducted after dietitians (*n*=18) were trained in EAT. Sessions were coded by trained, independent, coders using a study checklist, the Behaviour Change Counselling Index (BECCI) and items from the Cognitive Therapy Scale-Revised (CTS-R). Patient nutritional status was measured using the Patient-Generated Subjective Global Assessment (PGSGA).

**Results:**

Dietitian adherence to a written nutrition plan (*β*=7.62, 95% CI=0.65 to 14.58, *p*=0.032), dietitian adherence to behaviour change counselling (β=0.69, 95% CI =0.02 to 1.38, *p*=0.045) and competence in delivering behaviour change counselling (*β*=3.50, 95% CI =0.47 to 6.53, *p*=0.024) were significant predictors of patient nutritional status. Dietitian adherence and competence ratings were higher during consultations with intervention patients at greater risk of malnutrition.

**Conclusions:**

This study contributes new insights into the relationship between fidelity and treatment outcome by demonstrating that dietitian adherence and competence is greater when working with more challenging patients. This is likely central to the demonstrated success of the EAT intervention in reducing malnutrition and highlights the importance of ensuring that providers are adequately equipped to flexibly integrate intervention elements according to patient need.

**Trial registration:**

This study is a process analysis of a stepped-wedge randomised controlled trial prospectively registered on the Australian New Zealand Clinical Trials Registry (ACTRN12613000320752; Date of registration 21/03/2013).

**Supplementary Information:**

The online version contains supplementary material available at 10.1186/s13012-021-01118-y.

Contributions to the literature

- A central limitation of the complex behaviour change literature is that evaluations tend to focus on effectiveness and/or efficacy with comparatively less attention paid to key process variables including intervention fidelity.
- Within a real-world evaluation of a complex behaviour change intervention, we found evidence that dietitian adherence and competence is greater when working with more challenging patients.
- By addressing a longstanding limitation within the literature, this study makes an important contribution to the science of fidelity, including how dietitian adherence and competence may influence intervention outcomes within real-world clinical settings.

## Background

Fidelity is a multi-component and multidimensional construct [1] that is relevant to the design, conduct, evaluation and reporting of behavioural interventions [2–4]. When applied to the delivery of an intervention, fidelity refers to whether an intervention was delivered as intended (integrity) and the degree to which it is distinguishable from comparison conditions (differentiation [2, 3]). Key concepts of integrity include adherence (the degree to which an intervention was delivered as intended) and competence (the skill with which the intervention was delivered [5]). Accurate interpretation of treatment effects relies, in part, on evidence regarding treatment fidelity [6–8]. For example, demonstrating that an intervention was delivered with fidelity improves the confidence with which observed changes in patient outcome can be attributed to the intervention under evaluation [2, 3, 8]. Rigorous fidelity assessment is therefore critical for enhancing the external validity of a study [9].

Cognitive behaviour therapy (CBT) [10] and/or motivational interviewing (MI) [11] are commonly used evidence-based approaches for promoting change across a range of health behaviours [12–14]. These psychological approaches to health behaviour change can be defined as ‘complex’ due to the multiple interacting components employed [15]. Current understanding of complex behaviour change interventions is limited by inadequate consideration of treatment fidelity [16–18]. Systematic reviews and/or meta-analyses consistently demonstrate that fidelity assessment is rarely conducted [13, 19–25]. Furthermore, even when fidelity assessment is conducted, the resultant data (i.e. the level of adherence and/ or competence achieved) is rarely reported [20–24]. For example, in a recent review of MI for health behaviour change, of the 37 studies included, only 13 assessed fidelity and of those, more than half (53%) did not report the findings [21]. This means that, for the vast majority of evaluations, the concordance between the intended intervention and what was actually delivered is unclear. This compromises current understanding of whether and how complex behaviour change interventions affect change in patient behaviour. Secondary analyses are therefore recommended to explore the relationship between important process variables, such as fidelity, and treatment outcomes [26].

Intuitively, it may be reasonable to hypothesise that the more faithful a clinician is to delivering an intervention ‘as intended’ (i.e. greater levels of adherence and/or competence) the more beneficial the intervention will be. However, evidence regarding the relationship between fidelity and outcome is limited and complex [27, 28]. For example, in a seminal review of the health prevention literature, of the 162 publications included, only five examined the relationship between adherence and treatment outcome, and the presence and direction of effect varied within and between studies [29]. Similarly, adherence to the relational [21, 30, 31] and/ or technical [21, 31] components of MI has been linked to improved patient outcomes [21], worse patient outcomes [31] and no effect [21, 30, 31]. Greater attention to fidelity [1, 9], including the conduct of process analyses [26], is central to improving understanding of the relationship between fidelity and intervention effects.

Accordingly, we recently applied published recommendations [2–4] to the design and conduct of a comprehensive fidelity evaluation [32, 33] within a real-world multi-site stepped-wedge randomised controlled trial of ‘EAT: Eating as Treatment’. EAT is a complex health behaviour change intervention designed to reduce malnutrition in head and neck cancer (HNC) patients undergoing radiotherapy [34, 35]. Our findings demonstrated that the primary outcome of nutritional status (as measured by the Patient-Generated Subjective Global Assessment; PGSGA [36]) was superior for intervention relative to control participants (*β* = −1.53; confidence interval = −2.93 to −0.13 [34]). Furthermore, fidelity analysis [32] confirmed that (a) objective ratings of dietitian adherence and competence favoured intervention relative to control sessions [32], and (b) intervention sessions were clearly distinct regarding core motivational and behavioural intervention elements, whereas generic or ‘common’ intervention elements remained stable [32]. In light of this evidence for both intervention effects and intervention fidelity, we now take the next step [26] in the evaluation of the EAT intervention and conduct a process analysis.

### Objectives and importance

In the current paper, we explore the potential functional elements of EAT by examining the relationship between dietitian adherence and competence ratings and patient nutritional status in a randomly selected sample of intervention participants. Given the limited, and often contradictory nature of the literature, we make no predictions regarding the potential direction of effect. Rather, the current analysis is exploratory and aims to:

1. Examine the magnitude and direction of association between objective ratings of dietitian *adherence* to the EAT intervention and the nutritional status of intervention patients.
2. Examine the magnitude and direction of association between objective ratings of dietitian *competence* in delivering the EAT intervention and the nutritional status of intervention patients.

## Methods

### Setting and trial design

The current paper is derived from data collected during the intervention phase of the EAT clinical trial (ACTRN12614000876695; Date of registration April 21, 2013), conducted between 2013 and 2016 within five major Australian hospitals located in Queensland, Western Australia, Victoria and South Australia. This research was conducted in accordance with the National Statement on Ethical conduct in Human Research [37]. Further information regarding study methods and findings are available in published protocol and outcome papers from the fidelity evaluation [32, 33] and overarching clinical trial [34, 35].

#### Participants

Inclusion criteria [34, 35] for participants in the EAT clinical trial were aged 18 years or older, pathologically confirmed diagnosis of HNC, undergoing radiotherapy (definitive or post-operative) with curative intent and receiving a prescribed dose of at least 60 Gy and nodal irradiation. Participants were required to provide written, informed consent and be available for follow-up for at least 6 months.

#### Intervention providers

Eighteen oncology dietitians employed by the study sites were identified (via self and/or head of department selection) to undergo training in the EAT intervention. All intervention dietitians had attained bachelor level qualifications. Half (*n*=9) had completed postgraduate level training in nutrition and dietetics (including masters or postgraduate diploma in nutrition/dietetics). Experience working with HNC patients ranged from a few months to more than 20 years (mean = 3.38 years; mode = 1 year).

### Training

Training in EAT comprised a 2-day workshop, 1-day clinical ‘shadowing’, ongoing supervision and coaching (at least monthly) and a follow-up ‘booster’ workshop (comprising 1-day workshop, and 1-day shadowing). EAT training was delivered by the same facilitators (trial Clinical/Health Psychologists and authors BB, ALB and/or AKB) as part of an overall package designed to support systems level change [38].

### The EAT intervention

**Fig. 1:**
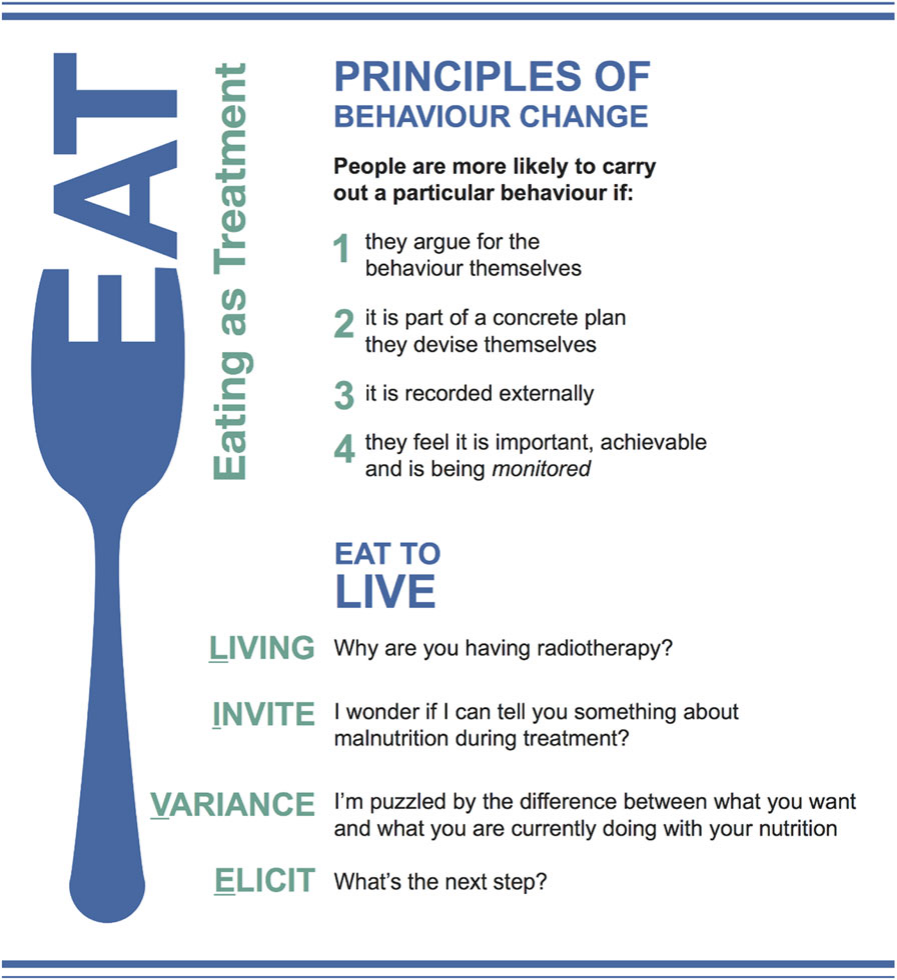
Eating As Treatment (EAT) intervention: key principles and prompts for clinicians

To support flexible integration throughout dietetic consultations (i.e. according to clinical presentation and/or patient need), EAT is informed by motivational and behavioural principles (Fig. 1).

MI is used throughout consultations to elicit and reinforce patient reason(s) for maintaining adequate nutrition, the performance of helpful nutrition-related behaviours and to collaboratively identify pertinent nutrition-related goals. A written nutrition planner is used to simplify, document, review and reinforce patient goals. An objective measure of nutritional status is provided via the conduct of a standardised nutrition assessment.

The “EAT to LIVE” conversation (see Fig. 1) integrates the aforementioned behaviour change principles and strategies. This conversation requires that dietitians (i) elicit patient motivation for undergoing radiotherapy treatment (i.e., survival); (ii) explicitly highlight the relationship between adequate nutritional status and radiotherapy treatment outcomes; (iii) explore the (in) consistency between patient behaviour, motivation to survive, nutritional status and requirements for maintaining an adequate nutritional status; and (iv) work towards developing a concrete plan.

### Assessment of fidelity of intervention delivery

#### Coding

Fidelity assessment in the EAT clinical trial was conducted using a randomly selected 20% sample of audio-recorded dietetic consultations conducted before (*n*=196) and after (*n*=194) training in EAT [32, 33]. In the current paper, we focus on the dietitian adherence and competence outcomes derived from the 194 randomly selected sessions conducted during the intervention phase of the EAT clinical trial. Audio-recordings were coded by an independent assessor (KB) blind to the schedule of training and intervention content. Each audio-recording was listened to from start to finish, and coding was based on the entire session. Inter-rater and intra-rater reliability was derived from a randomly selected 20% subsample of the coded intervention recordings (*n*=47). Inter-rater reliability was, on average, ‘substantial’ [39] (0.61), with a range from 0.24 to 1.0. Overall intra-rater reliability for both rater one (KB; 0.96, range 0.92 to 1.0) and rater two (KM; 0.94, range 0.84 to 1.0) was ‘almost perfect’ [39] (Supplementary Table 2).

### Measures

#### Adherence

##### Study checklist

Dietitian adherence to key elements of the EAT intervention was assessed using a checklist developed by the research team (see Table 1). Each of the items was rated as being present (‘yes’) or absent (‘no’).

**Table 1:** Study-specific fidelity checklist

	Yes	No
Practitioner discusses the adequacy of the patient’s energy intake		
Practitioner conducts a formal/standardised assessment to measure patient nutrition		
Practitioner discusses how eating/nutrition is an integral part of “radiotherapy” treatment**		
Practitioner encourages the patient to discuss their reason(s) for undergoing radiotherapy**		
Practitioner collaboratively develops a formal, written nutrition plan with the patient**		
Practitioner encourages the patient to discuss their progress towards the goals outlined on their written nutrition plan		

**Adherence to these items was found to be significantly greater during intervention relative to control sessions (*p*<.001) [32]

##### The behaviour change Counselling index (BECCI)

The 11-item BECCI [40] was used to assess dietitian adherence to MI. Each item is rated on a five-point scale (0=not at all, 1=minimally, 2=to some extent, 3=a good deal, 4=a great extent) and used to calculate an overall ‘practitioner score’.

##### The cognitive therapy scale–revised (CTS-R)

To assess the degree to which dietitians embodied the ‘spirit’ of intervention delivery (empathy, genuineness and warmth), we used the ‘interpersonal effectiveness’ item from the CTS-R [41]. This item is rated on a scale of 0 to 6, with higher ratings indicating greater expression of warmth, concern, confidence, genuineness and professionalism.

##### Competence

Due to the paucity of brief, validated tools available at study inception to assess competence in behaviour change counselling, the CBT competence item from the CTS-R [41] was modified to reflect competence in skills of behaviour change counselling. This item is rated on a scale of 0 to 6, with higher ratings indicating greater competence.

##### Primary patient outcome

Participant nutritional status was measured on four occasions (first and final week of radiotherapy, 4 and 12 weeks post-radiotherapy) using the Patient-Generated Subjective Global Assessment (PGSGA [42]). This instrument combines patient reported medical history (weight loss, nutrition impact symptoms, intake and functional capacity) and clinician examination (comprising diagnosis, age, metabolic stress and physical examination of fat, muscle stores and fluid status) [42]. Items are scored based on their likely impact on nutrition. The PGSGA can be used to calculate a numerical score (0–35), with higher scores indicative of elevated risk of malnutrition [42].

#### Statistics

Data analysis was conducted using the IBM Statistical Package for the Social Sciences (SPSS) for windows, version 26.0 (2019).

##### Predictor variables

To allow for evaluation of within-dietitian, between-patients effects of fidelity on treatment outcome; adherence and competence scores assigned to each consultation were used to generate average adherence and competence ratings for each dietitian sampled during the intervention phase. For the study checklist, dietitian adherence scores for each item were generated by calculating the proportion of their consultations coded ‘yes’ for that skill. The total BECCI scores assigned to each consultation were averaged to generate a mean BECCI adherence score for each dietitian. Average scores for the CTS-R interpersonal effectiveness item and CTS-R competence item were also produced to generate mean ratings of interpersonal effectiveness and competence, respectively, for each dietitian. For each predictor variable, intervention participants were assigned the score of the dietitian they saw in the sampled consultation(s). When an individual participant had sessions sampled from multiple dietitians, individual dietitian scores were averaged to produce an overall score for each of the predictors.

##### Analysis

Baseline characteristics of intervention participants (*n*=156) with at least one dietetic consultation randomly selected for inclusion in the fidelity sample (*n*=107) were compared to intervention participants not included in the fidelity sample (*n*=49) using independent samples *t* tests, chi-square or Fisher’s exact test, as appropriate. Linear mixed model regressions were used to analyse associations between the nine fidelity predictors and the dependent variable of patient nutritional status. Predictors were analysed separately to avoid interactions between them. The model utilised unstructured covariance and included the following: cluster level variable of site (to adjust for differences between hospitals), individual level variables of baseline PGSGA (to adjust for differences in baseline nutritional status), tumour site and stage (to adjust for any differences between participants’ nutritional status because of differences in malignancies), calendar time (to adjust for any background temporal effects that could have confounded the stepped-wedge design) and assessment interval (to adjust for differences in nutritional status through radiotherapy and recovery). The model included a random individual level intercept to account for the repeated measures on individuals over assessment moments and a random coefficient for assessment moment to allow for heterogeneity in subject specific trends. The alpha level was set to *p*=0.05.

## Results

### Sample

During the intervention phase of the EAT clinical trial, the 156 intervention participants attended a total of 1568 dietetic consultations. The coded sample of dietetic consultations was conducted by the 18 trained dietitians and two locum dietitians. The mean number of sessions sampled per dietitian was 9.7 (range = 1 to 43). 107 intervention participants (68%) were represented in the fidelity sample. The average number of coded sessions per participant was 2 (range = 1–6). The demographic and clinical characteristics of patients included in the fidelity sample were largely comparable to those who were not sampled, with the exception of fewer radiotherapy fractions received and poorer baseline nutritional status (Supplementary Table 1).

### Adherence and competence

Average dietitian adherence and competence ratings are presented in Table 2. The use of each study checklist item ranged from an average of 13 to 96% of each dietitians’ sampled intervention consultations. The mean overall BECCI practitioner score was 23.17, meaning that on average, each dietitian used behaviour change counselling ‘to some extent’ [43] with intervention patients. The average dietitian score on the CTS-R interpersonal effectiveness item was in the ‘expert’ [44, 45] range, meaning that intervention sessions were clearly delivered within the required ‘spirit’ (i.e. a high degree of empathy, warmth, genuineness and concern). The average dietitian score on the CTS-R competence item suggests that on average, intervention dietitians were ‘advanced beginners’ [44, 45].

**Table 2:** Average adherence and competence ratings for dietitians (*n*=20) whose consultations (*n*=194) with intervention patients (*n*=107) were sampled for fidelity analysis

	Proportion or M (SD)	Range
**Adherence**		
Study-specific checklist		
Reasons for RT	0.22	0 to 1.0
Eating as integral to radiotherapy	0.46	0 to 1.0
Nutrition plan	0.27	0 to 0.85
Review plan	0.13	0 to 0.50
Validated nutrition assessment	0.73	0.53 to 1.0
Adequacy of intake	0.96	0.80 to 1.0
Behaviour Change Counselling Index		
Overall Practitioner Score	23.17 (2.39)	17.52 to 27.45
‘Spirit’ of intervention delivery		
CTS-R Interpersonal Effectiveness Score	5.65 (0.55)	4.20 to 6.00
**Competence**		
CTS-R application of behaviour change counselling item	2.63 (0.61)	1.10 to 3.60

#### Relationship between fidelity and the nutritional status of intervention patients

Of the nine fidelity predictors (six study checklist items, BECCI practitioner score, CTS-R competence score, CTS-R interpersonal effectiveness score), three were found to be significantly related to the nutritional status of intervention participants (Table 3). From the study checklist, dietitian adherence to the nutrition plan was the only item that emerged as a significant predictor of patient nutritional status (*β* = 7.62, 95% CI = 0.65–14.58, *p* = 0.032). Dietitian adherence to behaviour change counselling (*β* = 0.69, 95% CI = 0.02–1.38, *p* = 0.045) and dietitian competence (*β* = 3.50, 95% CI = 0.47–6.53, *p* = 0.024) were also found to be significant predictors. Amongst intervention patients (i.e. whose nutritional status on average was found to be significantly better than that of controls), participants whose dietitians used either the nutrition planner or behaviour change counselling skills more frequently than other dietitians, or who had a dietitian who was more skilful in intervention delivery, demonstrated higher PGSGA scores (i.e. at greater risk of malnutrition).

**Table 3:** Relationship between fidelity predictors and the nutritional status of intervention participants

Fixed Effects	*β*	95% Confidence interval		*p*
		Lower	Upper	
**Adherence**				
Study-specific checklist				
Reasons for RT	−1.16	−6.96	4.64	0.69
Eating as integral to radiotherapy	0.93	−4.79	6.64	0.748
Nutrition plan	7.62	0.65	14.58	0.032
Review plan	−8.37	−23.88	7.15	0.287
Validated nutrition assessment	−2.93	−10.95	5.07	0.468
Adequacy of intake	7.15	−15.73	30.04	0.536
Behaviour Change Counselling Index				
Overall Practitioner Score	0.69	0.02	1.38	0.045
‘Spirit’ of intervention delivery				
CTS-R interpersonal effectiveness score	−1.98	−7.46	3.50	0.476
**Competence**				
CTS-R application of behaviour change counselling item	3.50	0.47	6.53	0.024

## Discussion

We found evidence that dietitian adherence and competence were inversely related to patient nutritional status in a sample of HNC patients enrolled in the intervention phase of the EAT clinical trial. Aligned with published differentiation outcomes [32], significant predictors of nutritional status only emerged from those fidelity outcomes that could be used to distinguish intervention from control sessions. Higher levels of dietitian adherence to the nutritional planner or MI were associated with significantly higher PGSGA scores (i.e. poorer patient nutritional status). Similarly, a significant relationship emerged between higher levels of dietitian competence and higher (worse) PGSGA scores for intervention participants. These findings are consistent with the inverse relationship detected between fidelity and treatment outcome within other evaluations of health behaviour change interventions [29, 31, 46], and the psychotherapeutic literature more broadly [27, 47].

How then, do we interpret these results within the context of the intervention being effective compared to randomised controls, and clinician adherence and competence being significantly higher amongst intervention dietitians? Taken together with evidence for null [21, 30, 31] or mixed [29] findings, it is clear that the relationship between clinician adherence, clinician competence and patient outcome is inherently more complex than the assumption of a necessarily positive relationship (i.e. higher adherence should optimise intervention efficacy). Although seemingly intuitive, this assumption does not account for a range of factors [26], including the complex interaction between patient and clinician behaviours that are central to MI informed interventions [11, 30, 48]. Of particular relevance to the current study is the potential impact of ‘challenging’ patients on therapist behaviour.

Two hypotheses have been proposed regarding the direction of this effect [49]. Firstly, therapist adherence and competence may be adversely affected [49]. For example, ‘behavioural resistance’ on the part of the client has been linked to lower levels of clinician adherence [50]. Similarly, patient resistance can elicit clinician confrontation [51] (a behaviour which runs contrary to the empathic, collaborative non-judgemental stance of motivational approaches), thereby undermining clinician competence. A second hypothesis [49], and one that is consistent with both the findings from the current study, and effective training in MI [11], is that adherence and competence are expected to increase during consultations with challenging patients. For example, patient resistance should elicit greater clinician use of MI, which in turn, provides a foundation for engaging patients in behaviour change strategies (e.g. nutrition planner). Under these circumstances, competence ratings are also likely to be greater due to clinician ‘responsivity’ [52] and patient complexity [53].

Consistent with this second hypothesis, an evaluation of motivational enhancement therapy (MET) [49] demonstrated that patient motivation was a significant predictor of within clinician variability in MET adherence. That is, when patients were less motivated to change their behaviour, clinicians were more likely to implement MET skills. Applied to the current trial, this finding is encouraging, since it suggests that EAT provided dietitians with tools to utilise with ‘hard to treat’ patients. Clearly, further research is warranted to identify and understand the relationship between patient and clinician variables, intervention delivery and treatment outcome.

Within the current analysis, two of the EAT intervention features previously shown to differentiate intervention from control sessions [32] (eliciting patient motivation for undergoing radiotherapy treatment; explicitly highlighting the relationship between adequate nutritional status and radiotherapy treatment outcomes) did not emerge as significant predictors of the nutritional status of intervention participants. On the one hand, this finding could be interpreted as evidence for the redundancy of these intervention elements. An alternate view is that since both intervention elements are grounded in established principles of behaviour change (i.e. amplifying the intrinsic value of the new behaviour and/or reducing the value of the old behaviour [54].), we expect that the answer lies in the complex nature of the EAT intervention. That is, by definition, the synergistic action of multiple, interacting components is expected to be greater than any single intervention component [26]. Building from the current findings, mixed methods research is now needed to specifically model and evaluate the complex pathways by which EAT may exert its effect, both in terms of intervention components and contextual factors [55].

### Strengths and limitations

This study makes an important contribution to the literature concerning complex behaviour change interventions and patient outcomes. To the best of our knowledge, this is the first real-world evaluation of the relationship between clinician adherence, competence and patient outcome within a tertiary care setting. The EAT intervention was delivered by a range of real-world clinicians within the context of every day practice. Patient nutritional status was assessed by independent, trained assessors [34] using a gold standard approach to nutrition assessment [36, 42]. Fidelity outcomes are derived from independent coding of a randomly selected sample of real-world consultations, by a trained coder blind to treatment allocation. Coder reliability was good, lending confidence that our findings reflect true treatment effects (i.e. rather than measurement error). Consistent with published recommendations our approach to analysing the relationship between fidelity and treatment outcome allowed us to control for temporal confounds and plausible third variables [27].

There are also a number of limitations to consider when interpreting results. First, findings are derived from a 20% sample of audio-recorded consultations conducted during the intervention phase of the EAT clinical trial. Although objective coding of real consultations is considered gold standard [2, 3, 56], the associated time and cost limits the number of sessions that can be coded [1]. Accordingly, although our methods lend confidence in the accuracy of our findings, we are restricted in our ability to definitively comment on clinician adherence and competence outside of the current sample of recordings. Recent advances in automated approaches to coding [57] may help to address this common limitation within the fidelity literature. Secondly, although subgroup analysis lends confidence in the representativeness of the sample, not all intervention participants were represented in the sample of intervention consultations randomly selected for fidelity assessment. Thirdly, fidelity of intervention delivery was operationalised according to observable skills, relative to the purpose or function behind using the skill. This is a common feature of tools used to evaluate clinician adherence and competence [53, 58]. However, given that intervention implementation is influenced by a range of clinician, patient and contextual factors [55], ongoing monitoring and assessment of clinician understanding of how an intervention works and their decision making process when it comes to intervention implementation is essential. In the current study, we utilised regular supervision and coaching to support theory informed treatment decisions [33, 35]. Future evaluations should also assess other important clinician (e.g. personality [59]), patient (e.g. change talk [30]) and contextual factors (e.g. organisational social context and readiness for change [60]) that may influence intervention delivery. Finally, the majority of our intervention providers were women, working with English speaking patients undergoing radiotherapy for HNC. Further research is therefore needed to understand whether the current findings generalise to male clinicians, non-English speakers and other patient groups at risk of malnutrition.

### Implications for research and practice

This paper addresses an important methodological limitation within the complex behaviour change literature. Improved understanding of the relationship between fidelity and treatment outcome represents an important research priority. This paper therefore makes an important contribution to the science of fidelity and the quality with which complex behaviour change interventions are evaluated and reported. The current findings also have direct implications for future training and supervision in the EAT intervention. For example, the utility of the nutrition planner and MI should be highlighted and efforts made to ensure that clinicians are equipped to competently deliver these skills to challenging patients. Conversely, efforts should be made to ensure that less challenging patients do not miss out on important intervention elements.

To optimise the effectiveness of complex behaviour change interventions more broadly, future research designed to explicitly manipulate and evaluate the interplay between patient and provider behaviours, intervention delivery and treatment outcome is needed. This research would also have important implications for intervention design, delivery and training—for example by providing evidence for patient ‘cues’ that providers can use to tailor and optimise intervention delivery and provider ‘cues’ that trainers can use to tailor and optimise training and supervision. Such ‘responsivity’ [52] is especially important for intervention delivery and dissemination within the dynamic context of real-world service provision [61].

## Conclusions

The current paper lends further support to the complex interplay between patient behaviour and clinician fidelity. Dietitians’ adherence to either MI or a nutrition plan and their competence were all found to be inversely related to patient nutritional status. Dietitians were especially likely to draw upon and skilfully execute these intervention components when working with challenging clients. This is likely central to the demonstrated success of the EAT intervention in reducing malnutrition and highlights the importance of ensuring that providers are adequately equipped to flexibly integrate intervention elements according to patient need. Identifying the clinician, patient and contextual factors that mediate and/or moderate the relationship between fidelity and treatment outcome represents an important challenge for future research.

## Supplementary Information

**Additional file 1: Table 1** Baseline Characteristics of Intervention Participants According to Whether or Not They Had Audio-recorded Dietetic consultations(s) Randomly Selected for Inclusion in the Fidelity Sample.

**Additional file 2: Table 2**. Inter- and Intra-Rater Reliability for Intervention Recordings.

## Data Availability

The datasets used and/or analysed during the current study are available from the corresponding author on reasonable request.

## Abbreviations

EAT: Eating As Treatment
HNC: Head and neck cancer
PGSGA: Patient-Generated Subjective Global Assessment
HREC: Human Research Ethics Committee
BECCI: Behaviour Change Counselling Index
CTS-R: The Cognitive Scale–Revised
MET: Motivational enhancement therapy
