# Supplementary material for: Is fidelity to a complex behaviour change intervention associated with patient outcomes? Exploring the relationship between dietitian adherence and competence and the nutritional status of intervention patients in a successful stepped-wedge randomised clinical trial of eating as treatment (EAT)
Source: Implement Sci. 2021 Apr 26;16:46. doi: 10.1186/s13012-021-01118-y (PMC8077889; doi:10.1186/s13012-021-01118-y)
Supplement: Supplementary file 1 — Additional file 1: Table 1 Baseline Characteristics of Intervention Participants According to Whether or Not They Had Audio-recorded Dietetic consultations(s) Randomly Selected for Inclusion in the Fidelity Sample. [file 13012_2021_1118_MOESM1_ESM.docx]

Additional File

Supplementary Table 1.

*Baseline Characteristics of Intervention Participants According to Whether or Not They Had Audio-recorded Dietetic consultations(s) Randomly Selected for Inclusion in the Fidelity Sample*

|  | Included in the Fidelity Sample | |  |  |
| --- | --- | --- | --- | --- |
|  | No  (n=49) | Yes  (107) |  |  |
|  | n(%) | n(%) | χ^2^ or Fisher’s Exact Test | p^a^ |
| Male | 35 (71) | 83 (77) | 0.69 | 0.407 |
| Aboriginal of Torres Strait Islander | 0 (0) | 3 (0.02) | --- | 0.552 |
| Marital Status |  |  | 3.85 | 0.255 |
| Married/ defacto | 26 (53) | 65 (60) |  |  |
| Separated/divorced/widowed | 15 (30) | 22 (20) |  |  |
| Single/ never married | 8 (16) | 15 (14) |  |  |
| Other | 0 (0) | 5 (4) |  |  |
| Highest Level of Education: |  |  | 2.50 | 0.271 |
| Primary School | 1 (2) | 1 (0.9) |  |  |
| High School | 22 (44) | 61 (57) |  |  |
| University/ vocational college | 26 (53) | 45 (42) |  |  |
| Tumour site |  |  | 5.81 | 0.293 |
| Nasopharynx | 3 (6) | 8 (7) |  |  |
| Oropharynx | 27 (55) | 61 (57) |  |  |
| Oral cavity | 9 (18) | 27 (25) |  |  |
| Larynx | 6 (12) | 9 (8) |  |  |
| Hypopharynx | 2 (4) | 0 (0) |  |  |
| Unknown primary | 2 (4) | 2 (2) |  |  |
| Tumour stage |  |  | 1.43 | 0.723 |
| I | 1 (2) | 5 (4) |  |  |
| II | 4 (8) | 13 (12) |  |  |
| III | 12 (24) | 20 (18) |  |  |
| IV | 32 (65) | 69 (64) |  |  |
| Dysphagia rating (CTCAE) |  |  | 0.34 | 0.988 |
| Absent | 34 (69) | 74 (69) |  |  |
| Symptomatic, able to eat | 7 (14) | 13 (12) |  |  |
| Symptomatic, altered eating | 6 (12) | 14 (13) |  |  |
| Severely altered eating | 2 (4) | 6 (5) |  |  |
| Life threatening | 0 (0) | 0 (0) |  |  |
| Concurrent chemotherapy | 41 (83) | 85 (79) | 0.17 | 0.683 |
| Postoperative radiation therapy | 14 (28) | 39 (36) | 0.85 | 0.356 |
| Prophylactic PEG | 13 (26) | 25 (23) | 0.16 | 0.692 |
| Prophylactic NGT | 2 (4) | 2 (2) | --- | 0.591 |
| Continuous Variables | Mean (SD) | Mean (SD) | 95% CI | p |
| Age | 60.46 (10.59) | 57.44 (10.71) | -0.62 to 6.66 | 0.103 |
| Prescribed radiation (Gy) | 68.28 (3.18) | 67.41 (3.48) | -0.26 to 1.99 | 0.131 |
| Fraction number | 34.25 (2.07) | 33.03 (4.89) | 0.11 to 2.33 | 0.031 |
| Depression (PHQ-9) | 3.29 (3.64) | 4.49 (4.31) | -2.53 to 0.14 | 0.079 |
| Quality of Life (EORTC) | 83.97 (15.07) | 82.19 (13.46) | -3.33 to 6.89 | 0.490 |
| Weight (kilograms) | 81.47 (17.96) | 83.66 (19.89) | -8.54 to 4.17 | 0.497 |
| Nutritional Status (PGSGA) | 4.49 (4.23) | 6.08 (5.18) | -3.15 to -0.03 | 0.045 |

^a^Equal variances not assumed
