# Supplementary material for: Is fidelity to a complex behaviour change intervention associated with patient outcomes? Exploring the relationship between dietitian adherence and competence and the nutritional status of intervention patients in a successful stepped-wedge randomised clinical trial of eating as treatment (EAT)
Source: Implement Sci. 2021 Apr 26;16:46. doi: 10.1186/s13012-021-01118-y (PMC8077889; doi:10.1186/s13012-021-01118-y)
Supplement: Supplementary file 2 — Additional file 2: Table 2. Inter- and Intra-Rater Reliability for Intervention Recordings. [file 13012_2021_1118_MOESM2_ESM.docx]

Additional File

Supplementary Table 2.

*Inter- and Intra- Rater Reliability for Intervention Recordings*

|  | | | Study Checklist | | | | | |  |  |  | BECCI |  |  | Cognitive Therapy Scale-Revised | |  | |
| --- | --- | --- | --- | --- | --- | --- | --- | --- | --- | --- | --- | --- | --- | --- | --- | --- | --- | --- |
|  | | | Eating as Integral to RT | Reasons for RT | Written Nutrition Plan | Written Nutrition Plan Reviewed | Validated Nutrition Assessment | Adequacy of Intake |  |  |  | Overall Practitioner Score |  |  | Competence | Interpersonal Effectiveness |  | |
| Inter-rater Reliability | | | | | | | | | | | | | | | | |  | |
| Cohen’s Kappa | | | .83  (Almost perfect) | 1.0  (Almost perfect) | .58  (Moderate) | .79  (Substantial) | 0.64  (Moderate) | 0.24  (Fair) |  |  |  | --- |  |  | --- | --- |  | |
| ICC Single Measures | | | --- | --- | --- | --- | --- | --- |  |  |  | 0.65  (Substantial) |  |  | 0.49  (Moderate) | 0.29  (Fair) |  | |
|  |  | Intra-rater Reliability (Coder One) | | | | | | | | | | | | | | |  |  |
| Cohen’s Kappa | | | 1.0  (Almost perfect) | 1.0  (Almost perfect) | 1.0  (Almost perfect) | .92  (Almost perfect) | .92  (Almost perfect) | 1.0  (Almost perfect) |  |  |  | --- |  |  | --- | --- |  | |
| ICC Single Measures | | | --- | --- | --- | --- | --- | --- |  |  |  | .95  (Almost perfect) |  |  | .97  (Almost perfect) | .96  (Almost perfect) |  | |
|  |  | Intra-rater Reliability (Coder Two) | | | | | | | | | | | | | | |  | |
| Cohen’s  Kappa | | | 1.0  (Almost perfect) | 1.0  (Almost perfect) | .95  (Almost perfect) | .94  (Almost perfect) | 1.0  (Almost perfect) | 1.0  (Almost perfect) |  |  |  | --- |  |  | --- | --- |  | |
| ICC Single Measures | | | --- | --- | --- | --- | --- | --- |  |  |  | .93  (Almost perfect) |  |  | .84  (Almost perfect) | .88  (Almost perfect) |  | |

*Note.* Inter-rater reliability (between the two coders) and intra-rater reliability (for each coder) for the study specific checklist was assessed using Cohen’s kappa. For the BECCI and CTS-R competence and interpersonal effectiveness items, inter-rater and intra-rater reliability were assessed using two-way mixed, absolute agreement, single measure intraclass correlation coefficients (ICC). All indices of inter-rater and intra-rater reliability were interpreted according to published guidelines^39^: 0.01 (‘poor’ agreement); 0.02-0.2 (‘slight’ agreement); 0.21 to 0.40 (‘fair’ agreement); 0.41 to 0.60 (‘moderate’ agreement); 0.61 to 0.80 (‘substantial’ agreement); 0.81 to 1 (‘almost perfect’ agreement).
